# Pathophysiology and Outcomes of Endothelium Function in Coronary Microvascular Diseases: A Systematic Review of Randomized Controlled Trials and Multicenter Study

**DOI:** 10.3390/biomedicines10123010

**Published:** 2022-11-23

**Authors:** Sanjeet Singh Avtaar Singh, Francesco Nappi

**Affiliations:** 1 Department of Cardiothoracic Surgery, Royal Infirmary of Edinburgh, Edinburgh EH16 4SA, UK; 2 Department of Cardiac Surgery, Centre Cardiologique du Nord of Saint-Denis, 93200 Saint-Denis, France

**Keywords:** endothelial functions, endothelium-derived relaxing factor, coronary microvascular dysfunction, INOCA, percutaneous coronary intervention

## Abstract

Background: Coronary macrovascular disease is a concept that has been well-studied within the literature and has long been the subject of debates surrounding coronary artery bypass grafting (CABG) vs. Percutaneous Coronary Intervention (PCI). ISCHEMIA trial reported no statistical difference in the primary clinical endpoint between initial invasive management and initial conservative management, while in the ORBITA trial PCI did not improve angina frequency score significantly more than placebo, albeit PCI resulted in more patient-reported freedom from angina than placebo. However, these results did not prove the superiority of the PCI against OMT, therefore do not indicate the benefit of PCI vs. the OMT. Please rephrase the sentence. We reviewed the role of different factors responsible for endothelial dysfunction from recent randomized clinical trials (RCTs) and multicentre studies. Methods: A detailed search strategy was performed using a dataset that has previously been published. Data of pooled analysis include research articles (human and animal models), CABG, and PCI randomized controlled trials (RCTs). Details of the search strategy and the methods used for data pooling have been published previously and registered with Open-Source Framework. Results: The roles of nitric oxide (NO), endothelium-derived contracting factors (EDCFs), and vasodilator prostaglandins (e.g., prostacyclin), as well as endothelium-dependent hyperpolarization (EDH) factors, are crucial for the maintenance of vasomotor tone within the coronary vasculature. These homeostatic mechanisms are affected by sheer forces and other several factors that are currently being studied, such as vaping. The role of intracoronary testing is crucial when determining the effects of therapeutic medications with further studies on the horizon. Conclusion: The true impact of coronary microvascular dysfunction (CMD) is perhaps underappreciated, which supports the role of medical therapy in determining outcomes. Ongoing trials are underway to further investigate the role of therapeutic agents in secondary prevention.

## 1. Introduction

In the last two decades, numerous publications have identified the pathogenetic mechanisms of endothelial dysfunction and anomalies of coronary vasomotor function and how they have a significant impact on cardiovascular disease [1,2,3,4,5,6,7,8,9,10]. In cardiac architecture, the coronary compartment with its circulatory function plays a key role in ensuring integrity in the role of a muscle pump through the maintenance of adequate coronary vasomotion. Disorders involving coronary vasomotion may be characterized by greater coronary vasoconstrictive reactivity at the epicardial and microvascular levels. Similarly, the impairment of endothelium-dependent and endothelium-independent coronary vasodilator capacity takes on crucial pathological significance, as does the enhancement of coronary vasodilatory potential related to changes in microvascular resistance caused by structural factors [1,2,3,4,5]. Several studies reported the role of endothelial dysfunction which has been substantially associated with the development and progression of macrovascular and microvascular coronary disease. However strong evidence suggests that Rho-kinase-induced myosin light chain phosphorylation leads to hypercontraction of vascular smooth muscle cells (VSMC) rather than endothelial dysfunction [1,2] as the former is the central mechanism of coronary spasm at epicardial and microvascular levels [6,7,8,9,10].

The following two fundamentally different types of coronary deficiency occur in humans: coronary macrovascular or microvascular disease. Both conditions can lead to ischemic heart disease and heart failure with or without preserved ejection fraction, representing the spectrum of coronary artery disease (CAD). Structural and functional abnormalities of “epicardial” coronary arteries allow the identification of coronary macrovascular disease. Although they have distinct causes, structural and functional deficiency may be immediately distinguished by means of coronary angiography performed by an interventional cardiology laboratory, which raises, once the diagnosis is reached, the choice between the percutaneous coronary intervention (PCI) or coronary artery bypass grafting (CABG). Alkhouli and colleagues established a trend on 12,062,081 revascularization hospitalizations based on the evaluation of characteristics and outcomes of patients undergoing coronary revascularization. Evidence suggested that significant changes occurred in the characteristics of patients undergoing PCI and CABG between 2003 and 2016. Patients with CAD suitable to receive coronary revascularization reported risk-adjusted mortality that was significantly reduced in individuals managed with CABG compared to those undergoing PCI [11,12].

Similarly, knowledge of the cause of structural and functional abnormalities of coronary microvasculature has important implication for the treatment of coronary microvascular dysfunction (CMD) that occur in many clinical settings. Important evidence has recently emerged from the PROMIS-HFpEF multicentre study in which the correlation between coronary microvascular dysfunction and heart failure with preserved ejection fraction (HFpEF) was evaluated [13,14,15,16,17,18,19]. Patients with HFpEF revealed an exertional hemodynamic abnormality [15] sustained by an elicited inflammatory response [16,17,18,19]. Again, structural and functional abnormalities of coronary microvasculature have been noted in ischemic heart disease [20,21,22,23,24,25,26,27], aortic stenosis [28], and even noncardiac disease, such as chronic inflammatory disorders [28,29,30,31,32,33,34,35] and liver diseases [36].

To the best of our knowledge, CMD is a pathophysiological entity, known as ischaemia and no obstructive CAD (INOCA), in which coexist ischaemia and no obstructive CAD are linked, the disease results in chest pain that develops over time, leading to further progression and is characterized by the presence or absence of coronary macrovascular disorder responsible to evoke an epicardial obstructive CAD [37,38,39]. Despite patients with INOCA from CMD experiencing steadily high prevalence and important prognostic course and consideration in both genders, it occurs, especially in women [20,21,22,23,24,25,26]. Of note that different subtypes of coronary vasomotion severity were recorded, even among asymptomatic patients, that impose consideration for the multiplicity of subclinical combinations, leading to myocardial ischaemia due to CMD [23,40,41,42].

Practice guidelines recommend consideration of PCI or CABG for patients with severe CAD despite best available medical therapy; however, emerging evidence does not specify whether standard surgical approach or percutaneous procedure because conclusive evidence is lacking to indicate which of these interventions is superior in the presence of coronary macrovascular or microvascular disease [43,44,45]. However, the benefits associated with endothelium-derived vasodilator agents have been established to play a key role in regulating vascular tone and preventing atherosclerotic progression. The improved outcomes using the left internal thoracic artery (LITA) graft are related to its superiority, which can be explained by the secretion of vasoactive agents. Similarly, the highest patency rates of LITA are due to its peculiar function of a “drug-eluting graft”. resulting in a beneficial effect on the downstream macro- and microvasculature [46].

A 2009 meta-analysis evaluating 10 trials that were conducted before 2006, included data from 7812 patients with dissimilar types of multivessel coronary disease. This report revealed that CABG and percutaneous coronary intervention (PCI) resulted in equivalent overall mortality at 5.9 years of follow-up (15% and 16%, respectively; *p* = 0.12) [47]. Recipients of CABG operations were less likely to undergo a repeat intervention as compared to those who were managed with PCI; nevertheless, the former were more likely to have a stroke. Importantly, this meta-analysis was affected by several limitations because large numbers of patients in whom CABG operation was known to improve survival both in patients with the coronary macrovascular disease or with CMD were lacking. Moreover, these trials resulted in scarce evidence of the cohort undergoing PCI on three-vessel disease or proximal left anterior descending coronary artery disease, diabetes, or left ventricular dysfunction. Additionally, in this cohort, no relevant data were produced regarding CMD [47]. More recently, evidence from the synergy between PCI with the Taxus and cardiac surgery (SYNTAX) trial suggested no significant difference in all-cause death between PCI using first-generation paclitaxel-eluting stents and CABG at 10 years. However, CABG provided a substantial survival benefit for patients in which three-vessel disease occurred. Of note, the best survival outcomes have not been demonstrated in patients with left main coronary artery disease.

We have been made aware of two randomized trials (ORBITA and ISCHEMIA) that compared medical management to PCI for stable CAD raising questions about the benefits of optimal treatment options between the two procedures and suggesting the importance of the coronary microvascular physiology, which an interventional approach could not enhance [48,49]. Of note that these trials are lacking results that directly address the role of coronary microvascular function, despite the substantial role of CMD, which is highly prevalent in patients with a wide spectrum of CAD, to the occurrence of residual cardiac ischaemia even after the successful coronary revascularization.

In this context, the endothelium can play a crucial role as evidenced by the molecules it produces in the regulation of the vascular tone. The endothelium synthesizes and liberates several derived relaxing factors (EDRFs), including nitric oxide (NO), endothelium-derived contracting factors (EDCFs), and vasodilator prostaglandins (e.g., prostacyclin), as well as endothelium-dependent hyperpolarization (EDH) factors [1,50,51]. The current practice demonstrates a clear contradiction between the proven benefits of coronary revascularization and the very limited improvement of endothelial dysfunction that can be related to decreased production or action of EDRFs or increased responses of EDCFs, initiating the step toward atherosclerotic cardiovascular diseases.

To encourage a larger spread of knowledge about the pathophysiology of atherosclerosis and CAD, and to provide a guide for clinicians, we herein argue on the current evidence basis on the role of different factors responsible for endothelial dysfunction leading to dubious benefits attributable to coronary revascularization. Recent RCTs and multicentre studies were discussed.

## 2. Methods

The rationale for the current study, analytic approach, and prespecified end points were settled up during the performance of a previous study-level systematic review focused on the role of endothelium-derived relaxing factors and endothelial function (doi:10.17605/OSF.IO/AQT2E). Data of the pooled analyses include research articles (human and animal models), CABG, and PCI randomized controlled trials (RCTs). Details of the search strategy and the methods used for data pooling have been published previously.

**Figure 1:**
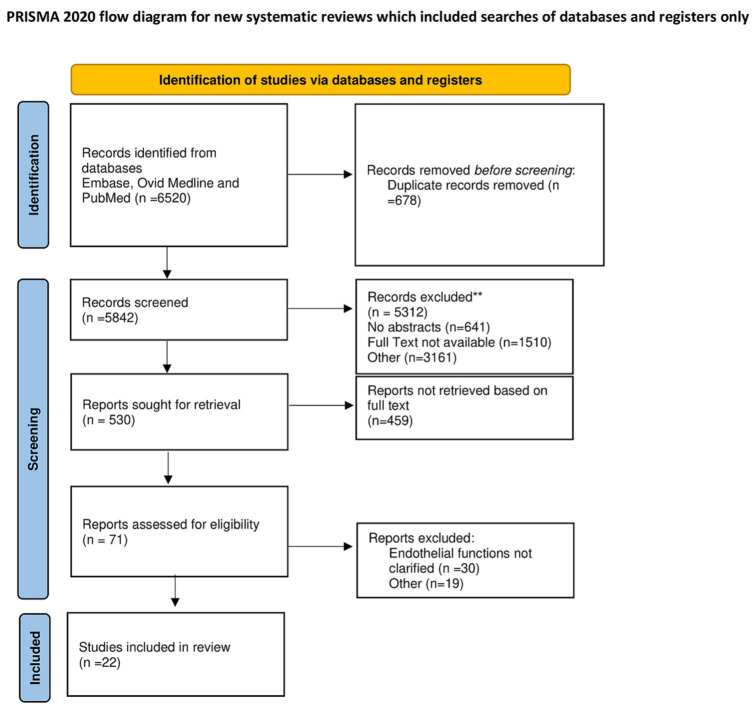
PRISMA flow diagram. For more information, visit: http://www.prisma-statement.org/ (accessed on 1 November 2022) ** Manuscripts that did not meet the criteria [52].

For the present analysis, the largest contemporary studies reporting the role of the endothelium in coronary microvascular diseases were included. Moreover, RCTs targeting endothelial function and coronary microvascular function were considered. Studies were stratified based on the endothelial modulation of vascular tone and coronary microvascular disease, both as regards the pathophysiological aspect and with regard to the most recent therapeutic approaches. The review was registered with the OSF register of systematic reviews (registration no is h5t2q) and followed the Preferred Reporting Items for Systematic Reviews and Meta-analyses (PRISMA) reporting guidelines. (Figure 1). Prisma check list in Supplementary Material. The project has been registered https://osf.io/h5t2q/?view_only=299975a535d64d83ad369baa1fb733e0 (accessed on 31 October 2022).

### Data Collection, Merging, and Endpoint of the Study

After the identification of eligible studies, SSAS and F.N obtained the necessary study-level collection data. Detailed specifications of core minimum de-identified data requirements were provided to each independent reviewer. The coordinating centre at Centre Cardiologique du Nord (F.N) received de-identified data that were verified for quality, completion, and coherency after evaluation of previous publications. As regard RCTs targeting endothelial function and coronary microvascular function data were controlled for missing values, intra-field data entirety, and inter-field inconsistencies both within RCT and across the RCTs. Discrepancies were worked out through direct consultation with independent reviewers. Endpoints included the contribution of blood vessel size-dependent EDRFS, the role of coronary microvascular disease, and the analysis of primary coronary microcirculatory dysfunction. Particular attention was given to endothelium-dependent CMD and advanced coronary atherosclerosis, as well as endothelium-dependent CMD and local low-shear stress. Additional analyses for modifiable risk factors for microvascular coronary heart disease and on promising tailored therapeutic strategies addressed to coronary microvascular function were also included.

Data elements from RCT, multicentre study, and RCT with upcoming results were then consolidated into a final database and included in Table 1.

## 3. Results

### 3.1. Endothelial Modulation of the Vascular Tone Insight on Blood Vessel Size-Dependent

#### Contribution of the EDRF

A wide range of reports has offered substantial evidence regarding the role of shear stress and various agonists in stimulating endothelial cells to synthesize and release different EDRFs that lead to the relaxation of the underlying VSMCs with consequential vasodilation [1,2,3,50,51]. There is broad consensus that three types of EDRF are key factors in determining endothelial modulation: vasodilator prostaglandins, NO, and EDH factors [1,2,3,50,51]. Animal studies addressing EDH-mediated relaxations revealed a well-defined production of cyclooxygenases and NO synthase inhibitors that are coupled with hyperpolarization of VSMCs located in the proximity of these molecular reactions. Some experimental studies have established the heterogeneous nature of EDH factors by reporting the specific characteristics relating to the different species and vascular beds of concern [53,54,55]. Greater production of epoxyeicosatrienoic acids, which constitute the fundamental metabolites of the arachidonic P450 epoxygenase sequential reactions were noted [56,57,58,59,60]. Moreover, the crucial role in electrical communication through gap junctions [61,62,63], and unequal production of K + ions [64,65,66], hydrogen sulphide (H2S) [67,68,69,70], monoxide of carbon (CO), [71,72] and endothelium-derived hydrogen peroxide (H_2_O_2_) [73,74] were observed. Evidence related to coronary endothelial vasomotion and addressing the physiological background of EDH-mediated relaxations for the demonstrated utility of long-term antispastic function, suggested a specific action of epoxyeicosatrienoic acids and Ca (2+) -activated K (+) channels in humans [75,76,77], canine [78], pig [79], and bovine coronaries [80]. In the porcine and bovine coronary endothelium, an increase in the level of K + ions was shown [81,82,83], while CO was detected in the rat coronary arteries [72]. On the other hand, at a physiological level, low concentrations of H_2_O_2_ derived from the endothelium have been reported in the coronary circulation of humans [84,85] and animals [86,87,88,89], respectively. Several pivotal independent studies reported the cardiovascular pleiotropic effects of H2S [53,90,91,92,93]. Chai et al. and Naik et al. have suggested that H2S has a comparable function to that of other gaseous mediators such as shear stress-mediated vasomotor control in coronary and mesenteric arteries [91,92], while Yang and colleagues demonstrated blood pressure lowering effects [93]. Similarly, Feletou and colleagues highlighted the significant anti-inflammatory and antioxidant properties [53].

**Figure 2:**
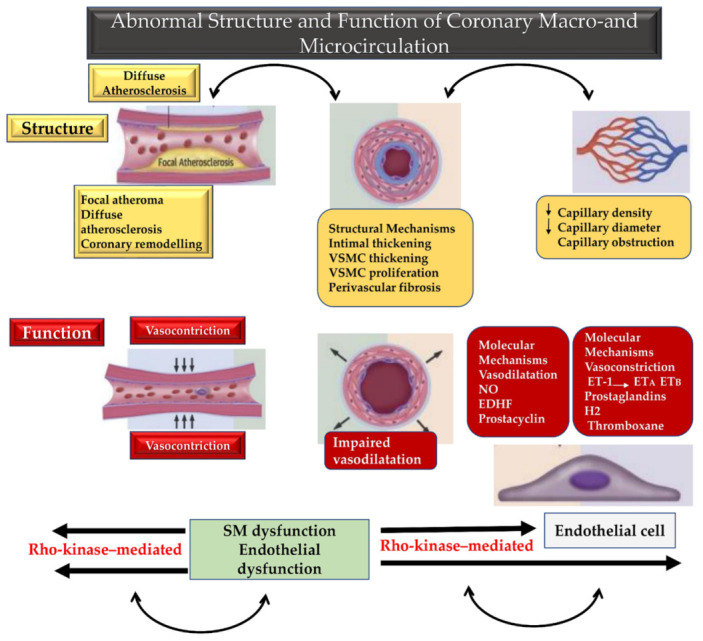
Blood vessel size-dependent endothelial modulation of vascular tone and Rho-kinase-mediated hypercontraction of vascular smooth muscles is depicted. Structural (yellow box) and functional (red box) anomalies are implicated in macrovascular and microvascular coronary dysfunction. Progressing from the superficial epicardium to the deep endocardium through the heart wall, large calibre and small calibre coronary vessels are involved in structural and functional disorders sustained by molecular and pathoanatomical mechanisms. EDRFs work to adjust the vascular tone that is substantially distinct from the size of blood vessels. Shear stress and various agonists lead to the stimulation of endothelial cells, which produce and release various EDRFs (vasodilator prostaglandins, NO, EDH) leading to relaxation of the underlying VSMCs and subsequent vasodilatation. A cascade process favours of the neighbouring VSMCs through EDH-mediated relaxations that is revealed in the presence of cyclooxygenase and NO. Abbreviations; EDH, endothelium-dependent hyperpolarization; EDRFs, endothelium derived relaxing factors; NO, nitric oxide; SMC, smooth muscle cell; VSMCs, vascular smooth muscle cells; ET1, endothelin 1; ETA, endothelin receptors type A; ETB, endothelin receptors type B.

The work of NO on vascular tone is mainly directed towards the large arteries located on the surface of the heart and identifiable with the epicardial coronary arteries. The support offered by the NO is reduced with the proportional reduction of the calibre of the target coronary artery, to determine a leap in the effect of mediated vasodilation between macro and micro coronary vessel circulation. On the contrary, the vasomotor effect sustained by EDH increases with the reduction in the calibre of the arteries, thus favouring vasodilation in the small resistance coronary branches of the subendocardial microcirculation [1,50,51,94,95,96]. Given the evidence that emerged, the most favourable target for the action of EDH is the distal coronary microcirculation where the small conduits are mostly interested in the functional variation of blood pressure and organ perfusion. A submaximal (>90%) distribution of the target coronary arteries to the effect of EDRFs induced relaxation is represented by essential vessel distribution of pre-arterioles (more than 100 μm in diameter) and arterioles (less than 100 μm), compared to only 5% of macrovascular epicardial coronary distribution [2,97]. This anatomical functional background related to the distribution of micro and macro coronary circulation, as well as to the effects of EDRFs, endothelin 1 (ET1), endothelin receptors type A (ETA) and type B (ETB), as well as prostacyclin and prostaglandins. Moreover, the effects of the former imply that the mechanisms of vasodilation by EDH-mediated responses become more important than NO-mediated coronary macrovessel relaxation. Again, several studies have suggested evidence of multiple mechanisms that are involved in EDH-mediated increased responses in small resistance arteries and report significant negative effects in terms of interactions between NO and several EDH factors [96,98,99,100,101]. Therefore, the treatment of patients with microvascular coronary diseases deserves particular attention from cardiologists as the anomalies of the coronary microcirculation are not detectable in routine coronary angiography Figure 2.

### 3.2. Evidence from Coronary Microvascular Disease Deploying

#### 3.2.1. Insights on Mechanisms, Prevalence, and Clinical Significance of CMD

**Figure 3:**
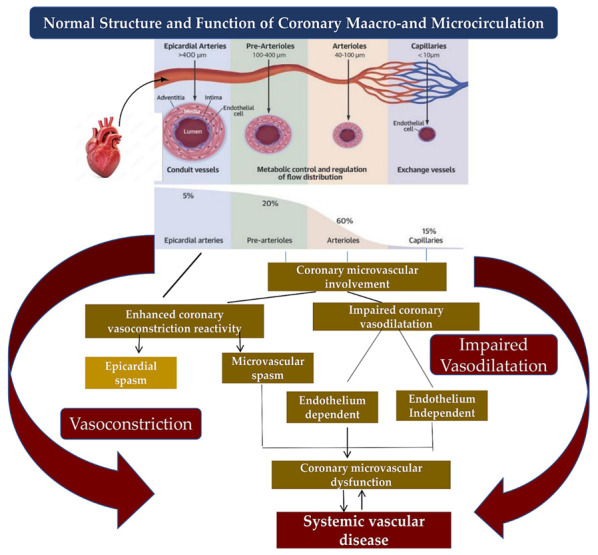
Mechanisms of coronary macrovascular and microvascular dysfunctions. Normal structure and function of coronary macro- and microcirculation is composed by epicardial arteries, pre-arterioles, arterioles, and capillaries. Epicardial spasm typically occurs in the epicardial arteries. On the contrary, coronary microvascular circulation can be subject to impaired coronary vasodilatation due to endothelial and non-endothelium-dependent factors and to microvascular spasm disorder. These phenomena lead to coronary microvascular dysfunction which supports a systemic vascular disease due to vasoconstriction or impaired vasodilatation.

The literature has multiple experimental and clinical evidence in the field of CMD honing onto the pathophysiological mechanism of cardiac ischaemia in patients with several cardiovascular illnesses associated with major clinical involvement [5]. Our knowledge suggests that several substantial structural alterations are decisive in causing CMD. These phenomena include increased coronary vasoconstrictive reactivity, typically manifested with coronary spasm both at the epicardial level and in the microvascular district, and reduced endothelium-dependent and endothelium-independent coronary vasodilator capacity. Similarly, increased coronary microvascular resistance caused by structural factors, such as those found in luminal narrowing, vascular remodelling, vascular rarefaction, and extramural compression, can lead to the onset of CMD. Although these alterations can manifest themselves in isolation or combine in various ways, nevertheless each of them can favour the onset of myocardial ischaemia [2,3,5,40,42,101,102,103,104,105]. Patients who experience coronary microvascular spasm record symptoms of angina with ischemic changes in the electrocardiogram. However, no epicardial spasm in response to the intracoronary challenge test with acetylcholine can be detected [106,107,108]. There are various molecular mechanisms that support coronary microvascular spasm, involving the central role worked by phosphorylation of the myosin light chain-mediated by Rho-kinase. [9]. Again, these mechanisms include an increase in the production of serotonin which is a mediator accompanying vasoconstrictive effects [109,110,111], of endothelin-1 [112,113,114,115,116], and neuropeptide Y [117,118]. Finally, inflammatory conditions in coronary microvasculature [119,120], with the consequent greater coronary vasoconstrictive reactivity, can induce coronary microvascular spasm. Recently, the role of MicroRNAs has been investigated for the interest driven by these small noncoding RNAs that regulate gene expressions through degradation or translational repression of mRNA. Evidence reported multiple regulatory roles exerted in the cardiovascular system by microRNAs [121,122,123]. Regarding CMD, it has been observed that microRNA-125a/b-5p is highly expressed in vascular endothelial cells and support an inhibitory effect on the expression of endothelin-1 [124]. Jaguszewski and colleagues reported reduced levels of 125a-5p microRNA which were associated with increased levels of plasma endothelin-1 in patients who experience Takotsubo cardiomyopathy. This alteration was suggestive of the development of coronary microvascular spasms [125]. Data from patients with CMD demonstrated that disease prevalence was unexpectedly high when CAD developed. AlBadri and colleagues reported that more than half of patients receiving invasive coronary angiography did not need significant coronary stenosis to assess for suspected coronary macrovascular lesions [126]. A previous study involving a large cohort of patients (*n* = 1439) revealed that approximately two-thirds of such patients with chest pain had angiographically normal coronary arteries or with nonobstructive lesions, CAD presented as an endothelium-dependent disorder or CMD disease independent of the endothelium, whereby the diagnosis was established through an invasive coronary reactivity test [23] Figure 3.

Patients who experienced this clinical condition are termed INOCA, a syndrome reflecting the well-specified role of CMD which is sustained by a different aetiology with symptoms and signs of myocardial ischaemia [37,38,39]. Several recently published studies using multimodal protocols to comprehensively study coronary physiology have revealed that a marked percentage of patients in whom INOCA occurs differ in the underlying form of the coronary microvascular disease [23,40,41,127]. In this regard, Suda and colleagues studied patients with vasospastic angina undergoing catheter-derived measures in the presence of a CMD. The authors revealed a significant 5% increase in the risk of major adverse cardiovascular events for every 1-point increase in the microcirculatory resistance index (IMR) [42]. Other independent studies performed over a 10-year period suggested that patients with INOCA, complicated by CMD, were associated with an increase in adverse cardiac events, including myocardial infarction, PCI or CABG procedures, cardiac death, and hospitalization due to the onset of unstable angina over time [128,129].

Cardiologists have several methods for assessing coronary microvascular function, with differences that fluctuate in cost, invasiveness, accessibility, quantifiable measures, and diagnostic accuracy. It should be known that the diagnostic accuracy of contemporary non-invasive stress tests is limited in CMD [13,92]. Instead, in order to identify patients with CMD, the complete invasive assessment of coronary vasomotor reactivity with intracoronary administration of acetylcholine, adenosine, and other vasoactive agents confers greater diagnostic reliability, associating feasibility and safety [23,40,130,131,132]. A clinical investigative approach aimed at the category of patients with CMD, in which coronary vasomotor disorders occur, can be fundamental to direct the most appropriate therapy. This type of approach is useful for providing health practitioners with valuable information to support decision-making and risk stratification beyond the assessment of traditional coronary heart disease risk factors.

#### 3.2.2. Lesson from CMD: A Systemic Vascular Disease Not Confined to the Heart

Recent reports have discussed how abnormalities attributable to coronary vasomotion often occur concomitantly with other peripheral endothelial diseases, giving CMD the role of systemic small arterial disease localized to the heart [133,134,135,136,137]. Ohura-Kajitani and colleagues simultaneously studied endothelial function in several vascular districts that included peripheral conduit, and resistance arteries in patients in whom VSA and microvascular angina occurred [134]. These cohorts of patients were assessed for CMD condition using the intracoronary acetylcholine test, which provokes coronary spasm [106,107,108,138]. In patients with microvascular angina, the main finding was the key role of bradykinin in inducing endothelium-dependent vasodilatation, which was almost absent in the arterioles of the fingertips [134]. The Ohura-Kajitani study demonstrated that mechanically induced digital vasodilation mediated by both NO and EDH were markedly impaired in patients with microvascular angina. This evidence explained that CMD is the manifestation of systemic vascular dysfunction not limited to the borders of the heart [134].

### 3.3. Impact of Primary Coronary Microcirculatory Dysfunction on Vulnerable Patients

#### 3.3.1. Simultaneous Occurrence of Endothelium-Dependent CMD and Advanced Coronary Atherosclerosis: A Dangerous Mixture

Godo and colleagues studied a series of patients with INOCA in whom endothelium-dependent CMD was associated with coronary atherosclerosis [139]. Evaluation of endothelium-dependent coronary vascular reactivity was achieved with graded doses of intracoronary acetylcholine. The criterion for defining an endothelium-dependent CMD was established in the presence of an increased percentage of coronary blood flow of less than 50% after the administration of acetylcholine [140,141]. Patients with VSA, which was defined as transient total or subtotal coronary artery occlusion where artery narrowing was greater than 90%, with chest pain and ischemic ECG changes in response to acetylcholine, were not allowed for evaluations. Their exclusion was due to the fact that acetylcholine has a limited effect when testing for endothelium-dependent CMD. It should be noted that acetylcholine does not have the effect of a pure endothelium-dependent agonist, but rather, its action can cause VSMC-dependent vasoconstriction in patients presenting VSA with associated greater coronary vasoconstrictive reactivity [1,2,3,50,51,131,132]. The main finding identified by Godo and colleagues was that, in patients with endothelium-dependent CMD, the presence of larger plaque burden and plaque volume was associated with more vulnerable plaque. These features of atheromatic plaque were obtained through intravascular virtual histology ultrasound [139]. Furthermore, the morphology of the plaque showed a greater volume of the necrotic nucleus combined with a higher frequency of thin-capped fibroatheroma, which is a peculiar characteristic of vulnerable plaques prone to rupture [139]. This evidence confirmed previous studies that a substantial association between endothelium-independent CMD and vulnerable plaque characteristics was demonstrated [142,143].

#### 3.3.2. Endothelium-Dependent CMD and Local Low Shear Stress

Shear stress substantially works to confer fundamental physiological stimuli that lead endothelial cells to synthesize and liberate EDRFs, thus maintaining vascular homeostasis. Conversely, the effect of established oscillatory stress or low shear stress can disrupt the flow on the coronary artery wall to determine the local progression of atherosclerotic coronary plaque. The result of this process is endothelial and VSMC proliferation, inflammation, lipoprotein uptake, and leukocyte adhesion [144,145].

It should be emphasized that previous studies have suggested the crucial role of altered shear stress that induces a detrimental effect on the coronary artery wall by promoting the local progression of atherosclerotic coronary plaque [146,147]. Furthermore, Siasos and colleagues investigating coronary endothelial shear stress showed a decrease in coronary blood flow variation in response to the decrease in acetylcholine [148]. This evidence clearly suggests that endothelium-dependent CMD is implicated in the progression of coronary atherosclerosis, very likely mediated by low endothelial shear stress [148,149].

#### 3.3.3. Vulnerable Microcirculation Opinion

The above evidence paves the way for two pivotal concepts in the CAD landscape: patients considered with primary coronary microcirculatory dysfunction [150] and those defined as vulnerable patients [151,152]. Patients who experience chest pain but who do not show angiographic abnormalities are often included in treatment programs that are marked by underdiagnosis. No therapeutic intervention or follow-up is recommended for this cohort of patients, because they are categorised as patients with coronary arteries without target lesions. Rather, CMD patients may be considered a more vulnerable category to developing coronary atherosclerosis and thus may be at risk of experiencing future coronary events [127].

## 4. A look at the Clinic and Therapy

### 4.1. Smoking and Vaping as a Risk Factor for Coronary Macrovascular and Microvascular Diseases

Consolidated experiences have identified cigarette smoking as one of the most traditional risk factors for the development of coronary atherosclerotic disease. Cigarette smoking also plays a major role as a negative prognostic factor for VSA [153,154,155,156], to the extent that patients with VSA who stop smoking experienced a drastically significant improvement in symptoms associated with improved prognosis [153,154]. The adverse effects due to cigarette smoke is related to the formation of superoxide anions that can lead to oxidative degradation of NO which is more rapid. Furthermore, these molecules are directly involved in cell damage by promoting vascular inflammatory responses and sustaining an exacerbated coronary contraction [157,158]. Newly, the increase in the use of vaping products have been suggested as another cause supporting the pathogenesis of macrovascular and microvascular diseases [159,160,161,162]. There is no doubt that the smoke produced by mentholated cigarettes has the same effect in reducing coronary flow reserve as that exercised by normal cigarettes. The role that the flavouring additives present in electronic cigarettes in causing endothelial dysfunction is well-known, supporting vascular inflammatory responses and oxidative stress, to decrease the bioavailability of NO [159,160,161].

A discrepancy exists regarding the effects caused by e-cigarette smoking that support an increase in the acute vasoconstrictor response at the level of the microcirculation. The latter is in contrast to an enhancement in the index of microvascular endothelial function, indicated as an index of reactive hyperaemia (RHI), which unexpectedly increases promptly after using the electronic cigarette [162].

An RCT for the assessment of pro-atherosclerotic effects of smoking was drawn to study the acute effects of vaping e-cigarettes and heat-not-burn cigarettes on coronary vasomotor function. The study is focused on the use of invasive coronary reactivity tests, including coronary flow reserve, fractional flow reserve, and the instantaneous wave-free ratio [163,164]. The results that will emerge from this study will provide further evidence regarding the effects of new smoking market products as detrimental factors on coronary macrocirculation and microcirculation [163,164].

### 4.2. To Exceed or Not in the Treatment with NO?

The survival benefits associated with the use of nitrates as a NO donor for the treatment of the acute phase of ischemic heart disease and heart failure were established in a landmark paper published in The Lancet in 1879 [165]. We have described above the emergent role of CMD that can occur in patients with various cardiovascular diseases, including obstructive CAD, who received successfully CABG or PCI intervention [48], INOCA [39], VSA [42], and HFpEF [13,16,17]. It is important to underline that although the increase in NO-mediated vasodilation through the additional administration of NO could have beneficial effects on these patients, the data that emerged contrasted with the results achieved after systemic and long-term administrations of the medicament. Patients with residual microvascular ischaemia treated with nitrates gave responses that were unexpectedly neutral or even harmful despite the success of PCI [166], myocardial infarction [167], VSA [168], and HFpEF [169,170]. The suggested findings explain the potential damage of NO therapy and the need for researchers to focus on therapies to avoid excessive use of NO. Similarly, the paradoxical effect caused by the targeted use of NO could be explained by the occurrence of possible nitrosative stress, determined by an excessive amount of supplementary NO [171,172]. Extensive evidence has reported significant negative interactions between NO and various EDH factors [96,99,100] and an imbalance between EDH and NO responses on vascular tone has been described.

Specifically, EDH exerts a crucial effect on coronary vascular resistance, which is predominantly determined by coronary microcirculation [97], by favouring vascular tone-mediated responses that overcome the opposing NO-mediated relaxation responses. It is therefore important to consider the contribution of NO and EDH factors dependent on blood vessel size in the treatment of CMD.

Given the evidence provided, it has been observed that the use of intracoronary nitroglycerin does not increase coronary blood flow [139,173]. From this, considering the mechanisms that support the anomalies of coronary vasomotion, it is important to establish specific indications and consider possible contraindications to chronic supplementation of NO. Given these observations, it is a priority to personalize the most appropriate treatment that is widely reported with a good evidence base. [130,174,175].

### 4.3. An Overview of Clinical Trials Suggesting an Approach Focused on Endothelial Function and Coronary Microvascular Function

**Figure 4:**
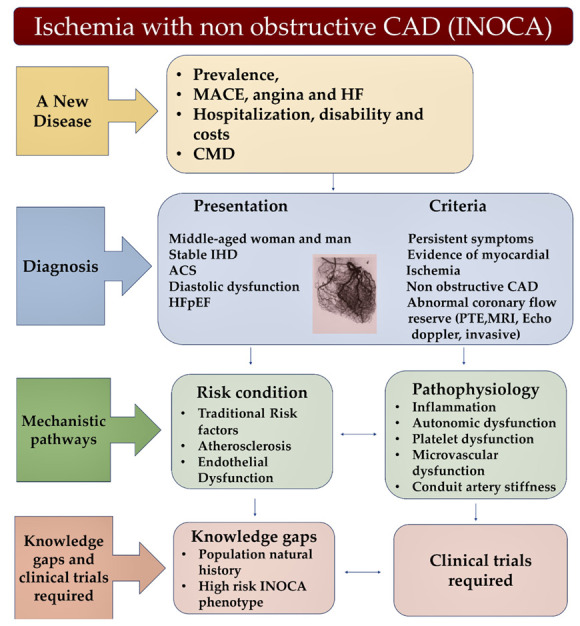
Ischaemia with nonobstructive CAD also called INOCA is a new disease (yellow box) characterized by a specific diagnosis accompanied by typical presentation and criteria (blue box). Risk condition and pathophysiology (green box), as well as an improvement of knowledge (brown box) are of primary importance for the management of INOCA. Abbreviations; IHD; ischaemic heart disease; MRI, Magnetic Resonance Imaging; positron emission tomography; other abbreviations in other figures and table.

Given the importance of endothelial function in the clinical setting, its evaluation appears crucial as an excellent surrogate indicator of cardiovascular risk. Therefore, it was taken into consideration during the diagnostic process of patients with CAD [176]. Practitioners and cardiologists use altered flow-mediated dilation (FMD) of the brachial artery and digital RHI in peripheral arterial tonometry, which are both substantially correlated with future cardiovascular adverse events in patients with CAD [177,178,179,180,181]. In this regard, a doubling of the risk of adverse cardiovascular events following a reduction in the standard deviation of FMD or RHI was reported by Matsuzawa and colleagues [182]. Although there are some points of convergence between macrovascular and peripheral microvascular endothelial function in supporting CAD, the role of FMD in macrovascular endothelial function has been well established while RHI intervenes preferentially in peripheral microvascular function. However, it should be noted that both indices are often altered in patients with CMD [183,184], for which the systemic nature of the disorder appears strongly suggestive (Figure 4).

The use of statins is currently recommended by the European Society of Cardiology guidelines for all patients with chronic coronary syndromes, including those with CMD [161]. The same guidelines state that the use of beta-blockers, angiotensin-converting enzyme (ACE) inhibitors, and statins are indicated for patients with reduced coronary flow reserve or an increased index of microcirculatory resistance with associated negative acetylcholine provocation test. This cohort of patient suffers from reduced coronary vasodilator capacity, while calcium channel blockers (CCBs) and long-acting nitrates are suggested for patients with coronary microvascular spasms [185]. Furthermore, previous studies on animal models have emphasised that the use of ACE inhibitors can offer an enhancement of the endothelium-dependent release mechanisms mediated by both NO and EDH factors in the coronary circulation [186,187].

These findings have paved the way for the implementation of a targeted therapeutic approach [130]. Numerous promising clinical trials are underway addressing stratified medical treatment guided by coronary reactivity testing and endothelial function-guided management, which may prove to be useful in patients with CMD. Of note, three independent RCTs deserve attention. The former is the multicentre, prospective, randomised, blinded clinical study, Women’s Ischaemia Trial to Reduce Events in Nonobstructive CAD (WARRIOR) (NCT03417388), which enrols 4422 patients, currently in progress. The aim of the study is whether intensive medical treatment with the administration of high-intensity statins, combined with the maximum tolerated doses of ACE inhibitors/angiotensin receptor blockers and aspirin use can reduce the risk of MACE in female patients with symptoms and/or signs of myocardial but nonobstructive ischaemia CAD [188].

The second is a large-scale randomized clinical trial, the Endothelial function-guided management in patients with Nonobstructive Coronary Artery Disease Study (ENDOFIND), which is currently recruiting. The trial, which was designed considering the specific category of patients in which CMD is highly prevalent, is aimed at evaluating whether peripheral endothelial function-guided early aggressive management can reduce the risk of MACE in patients with nonobstructive CAD. Patients enrolled have lifestyle management, optimal blood pressure, and glycemic control, and the intensive use of statins and CCBs as inclusion criteria [165]. Completion of these RCTs is expected by the end of 2022 and should offer crucial evidence on the management of patients with CMD [189].

Finally, we are awaiting the results of the Ticagrelor and preconditioning in patients with stable coronary artery disease TAPER-S RCT, which will be completed at the end of November and should provide some interesting answers. The study enrolled patients with stable multivessel CAD undergoing staged, fractional flow reserve-guided PCI. In these, the pleiotropic effects of an oral, direct-acting, reversibly binding, P2Y12 antagonist ticagrelor drug on ischaemic preconditioning and coronary microvascular function were evaluated [190].

## 5. Conclusions

Epicardial coronary arteries located on the surface of the heart in particular are susceptible to the interplay between the vasodilator prostaglandins, NO, and EDH factors. The true impact of CMD is perhaps underappreciated, which supports the role of medical therapy in determining outcomes. Ongoing studies are needed to further understand the role of secondary prevention, guided by invasive coronary tests, to provide robust evidence for the management of CMD and further identify the benefits afforded by CABG in treating CMD alongside coronary macrovascular disease.

## Figures and Tables

**Table 1:** Characteristics of the included studies.

Author/Year	Study Period	Total Number	Cohort (N)				Aims	Type	Findings
Mohri et al. (2003) JACC [9]	1999–2000	18	Saline		Fasudil 13		Role of Rho-kinase	RCT	Fasudil ameliorates myocardial ischaemia in patients with coronary microvascular spasm by mean of the inhibition of Rho-kinase
Masumoto et al. (2002) Circulation [10]	1999–2000	20	Saline 5		Fasudil 15		Role of Rho-kinase	RCT	Fasudil was effective in preventing ACh-induced coronary artery spasm and resultant myocardial ischaemia in patients with vasospastic angina.
Kataruka et al. (2020) JAHA [11]	2005–2017	215 066	PCI 178 474		CABG 36 592		Whether 30 days death ratio increased in PCI vs. CABG	Multicentre COAP database	Clinical acuity increased for patients treated with PCI rather than CABG with increased use of PCI instead of CABG. PCI 30 days death ratio increased (0.98 vs. 1.19, *p* < 0.0001). CABG decreased (1.21 vs. 0.74, *p* < 0.0001)
Alkhouli et al. (2020) JAMA [12]	2003–2016	12 062 081	PCI 8 687 338		CABG 3 374 743		Whether death increased in PCI vs. CABG	Multicentre observational	Risk-adjusted mortality decreased significantly after CABG but not after PCI. mortality increased in PCI (22.8% to 53.1%) decreased in CABG (5.6% to 3.4%)
Shah et al. (2018) EHJ [13]	2015–2018	263 202 HFpEF	CMD absent 51		CMD present 151		Whether CMD is higher in HFpEF.	Prospective Multicentre PROMIS-HFpEF	High prevalence of CMD (151) in HFpEF [75% (95% confidence interval 69–81%)] in the absence of unrevascularized macrovascular CAD. Smoking *p* = 0.0006 and atrial fibrillation *p* = 0.004 in CMD
Hage et al. (2020) J Card Fail [14]	2015–2018	263	CMD absent 51		CMD present 151		To determine association of CMD with hospitalization and mortality in HFpEF.	Prospective Multicentre PROMIS-HFpEF	CMD was independently associated with primarily CV- and HF-specific events.
Ahmad et al. (2021) Eur J Heart Fail [15].	2010–2019	51	HFpEF 22		No/HFpEF 29		Whether exist difference in CFR between HFpEF and No/HFpEF	Prospective Multicentre	Coronary microvascular function is inversely associated with filling pressures. HFpEF was associated to lower CFR (2.5 ± 0.6 vs. 3.2 ± 0.7; *p* = 0.0003)
Dryer et al. (2018) Am J Physiol Heart Circ Physiol [17]	2015–2017	44	HFpEF 30		No/HFpEF 14		To evaluate the incidence of coronary microvascular dysfunction in HFpEF	Prospective Multicentre USA	HFpEF had more abnormalities of coronary flow and resistance than to No/HFpEF
Suhrs et al. (2020) PLoS One [18]	2012–2017	431	336 women CAD 180 Db 156 nDb		95 controls		To evaluate if subclinical inflammation is associated with non-endothelial dependent CMD and diastolic dysfunction.	Prospective iPOWER study	Inflammatory biomarkers increased to both CMD and E/e’ diastolic dysfunction.
Yang et al. (2020) Eur J Heart Fail [19]	1993–2015	162	HFpEF 115		HFnpEF 47		To evaluate the role of ED and EI mechanism In HFpEF	Observational Multicentre	HFpEF was associated to CMD due to ED and EI mechanisms. Worse diastolic dysfunction is higher in HFpEF and EI-CMD.
Von Mering et al. (2004) Circulation [20]	1996–2000	† 163	No CAD 74	Minimal CAD 49	Severe CAD 40		Whether Ach influence vasomotion response in woman	RCT WISE	In woman impaired coronary vasomotor response to Ach. Ach independently linked to adverse cardiovascular outcomes (less time free from cardiovascular events (*p* = 0.004)
Murthy et al. (2014) Circulation [21]	2006–2010	1218	Men 405		Women 813		Whether there is a relative extent to which CMD affects both genders	Retrospective	CMD is not a uniquely female disorder and identifies men and women at increased clinical risk. CFR was a powerful incremental predictor of MACE (hazard ratio 0.80 [95% CI 0.75–086] per 10% increase in CFR; *p* < 0.0001)
Sara et al. (2015) JACC Cardio Int [23]	1993–2012	1439	CBFAch+, CFRAdn+ 520	CBFAch-, CFRAdn+ 478	CBFAch+, CFRAdn- 173	CBFAch−, CFRAdn- 268	Assessment of the prevalence of CM abnormalities in patients presenting with chest pain and CAD	Prospective	Patients with chest pain and nonobstructive CAD have high prevalence of CM abnormalities
Aziz et al. (2017) JACC [24]	2007–2014	1379	Male 573		Women 806		Determine sex differences of vasomotor dysfunction in a European population	Prospective	Female patients have a higher prevalence of vasomotor dysfunction (especially CMD) compared with male patients. Odds ratio 4.2 (95% confidence interval: 3.1 to 5.5; *p* < 0.001) and 2.3 (95% CI: 1.7 to 3.1; *p* < 0.001)
Motiejūnaitė et al. (2020) EHJ [25]	1995–2008	* 22 523 γ26 376	Male 12 589		Women 9933		Evaluate the association of sex and 1-year all-cause mortality in patients with AHF in various regions of the world.	Comparative Study	Globally women with AHF have a lower 1-year mortality and less evidenced-based treatment than men. (HR 0.86 (0.79–0.94), *p* < 0.001 after adjustment)
Schroder et al. (2021) EHJ [26]	2003–2008	All women 1681	CFVR < 2.25 723		CFVR ≥ 2.25 958		Whether assessment of CMD predicts adverse outcome in women with angina and no obstructive CAD.	Prospective iPOWER	CFVR by echocardiography is predictive of adverse outcome in women with angina and no obstructive CAD (HR 1.07; 95% CI 1.03–1.11)
Kakuta et al. (2021) JAHA [29]	2015–2018	67	IBD 37		Control 30		To investigate the presence and severity CMD in IBD	Retrospective	IBD is associated with CMD, which improved after surgical resection of diseased intestines.
Lee et al. (2015) Circulation [40]	2007–2012	139	Men 32		Woman 107		To investigate angina in symptomatic patients with nonobstructive CAD by using Ach, IMR, CFR, FFR and IVUS	Prospective	High rate of patients with angina in the absence of obstructive CAD have occult coronary abnormalities
Ford et al. (2019) Circ Cardiovasc Interv [41]	2016–2017	391	INOCA 195		CAD 206		To determine microvascular and vasospastic angina INOCA	RCT	Higher rate (3/4) of INOCA reveal coronary vasomotion disorders including microvascular and vasospastic angina
Suda et al. (2019) JACC [42]	2014–2017	187	Men 113		Woman 74		To evaluate the coronary functional abnormalities in both epicardial and microvascular coronary arteries in patients with angina and INOCA.	RCT	Patients with angina and INOCA have both epicardial coronary spasm and increased microvascular resistance. Worse prognosis and Rho-kinase activation may be involved.
Al-Lamee et al. (2018) Lancet [48]	2014–2017	200 Medically treated angina	PCI 105		Placebo 95		To evaluate efficacy of PCI on stable angina	RCT ORBITA	PCI did not increase exercise time (PCI minus placebo 16·6 s, 95% CI -8·9 to 42·0, *p* = 0·200).
Maron et al. (2020) NEJM [49]		5179	PCI plus OMT		OMT alone		Whether PCI plus OMT is effective compared OMT alone.	RCT ISCHEMIA	PCI is not effective in patients with stable coronary disease and moderate or severe ischaemia. At 5 years, (death, MI, Re-hosp) was 16.4% and 18.2%, respectively (difference, −1.8 percentage points; 95% CI, −4.7 to 1.0)

Abbreviations; Ach, acetylcholine; Adn, adenosine; CAD, coronary artery disease; CBF, coronary blood flow: CFR; coronary flow reserve; CFVR, coronary flow velocity reserve; CI, confidence interval; CM, coronary microvascular; CMD, coronary microvascular dysfunction; COAP, Cardiac Care Outcomes Assessment Program; ΔCSA, coronary cross-sectional area; E/e’; indicator of diastolic function; ED- CMD, endothelium dependent coronary microvascular dysfunction; EI- CMD, endothelium independent coronary microvascular dysfunction; EF, endothelial function; FFR, fractional flow reserve; HFpEF, heart failure with preserved ejection fraction; HFnpEF, heart failure with non-preserved ejection fraction; IBD, inflammatory bowel disease; IMR, microvascular resistance; INOCA, no obstructive coronary artery disease; IVUS, intravascular ultrasound; LVDDP, LV end-diastolic pressure; MPBIMR; myocardial perfusion based non-invasive index of microcirculatory resistance; WISE, Women’s Ischaemia Syndrome Evaluation. † Total Wise enrolment 936 women; * GREAT registry; γ OPTIMIZE-HF.

## Data Availability

Not applicable.

## Supplementary Materials

The following supporting information can be downloaded at: https://www.mdpi.com/article/10.3390/biomedicines10123010/s1, Supplementary Table S1: Prisma checklist 2020, reference [52] is cited in supplementary materials.

## Abbreviations

CAD: coronary artery disease; CFR: coronary flow reserve; CMD: coronary microvascular dysfunction; CMP: cardiomyopathy.
